# Management of immune checkpoint inhibitor‐related dermatologic adverse events

**DOI:** 10.1111/1759-7714.13275

**Published:** 2019-12-09

**Authors:** Xiaoyan Si, Chunxia He, Li Zhang, Xiaowei Liu, Yue Li, Hanping Wang, Xiaoxiao Guo, Jiaxin Zhou, Lian Duan, Mengzhao Wang, Li Zhang

**Affiliations:** ^1^ Department of Pulmonary and Critical Care Medicine Peking Union Medical College Hospital Beijing China; ^2^ Department of Dermatology Peking Union Medical College Hospital Beijing China; ^3^ Department of Clinical Laboratory Peking Union Medical College Hospital Beijing China; ^4^ Department of Ophthalmology Peking Union Medical College Hospital Beijing China; ^5^ Department of Gastroenterology Peking Union Medical College Hospital Beijing China; ^6^ Department of Cardiology Peking Union Medical College Hospital Beijing China; ^7^ Department of Rheumatology Peking Union Medical College Hospital Beijing China; ^8^ Department of Endocrinology Peking Union Medical College Hospital Beijing China

**Keywords:** Dermatologic toxicities, immune checkpoint inhibitor (ICI), immune‐related adverse events (IrAEs)

## Abstract

Immune checkpoint inhibitors (ICIs) have revolutionized cancer treatment. The unique spectrum of immune‐related adverse events (IrAEs) may occur during treatment. Dermatologic toxicities appear to be one of the most prevalent immunotherapy‐related adverse events. The most common symptoms are maculopapular rash and pruritus. Serious dermatologic toxicities including Stevens‐Johnson syndrome (SJS), toxic epidermal necrolysis (TEN), and drug reactions with eosinophilia and systemic symptoms are rare. In this review, we summarize guidelines of management of immunotherapy‐related toxicities, case reports, and proposed treatment recommendation.

## Key points

This review will enable clinical oncologists to recognize, diagnose, and manage immune checkpoint inhibitor‐related dermatologic adverse events.

## Introduction

Immune checkpoint inhibitors (ICIs) have shown antitumor activity in various malignant tumors, such as melanoma, non‐small cell lung cancer (NSCLC), renal cell cancer, Hodgkin's lymphoma, etc. ICIs include cytotoxic T lymphocyte associated antigen‐4 (CTLA‐4: monoclonal antibody ipilimumab), programmed cell death protein (PD‐1: monoclonal antibody nivolumab, pembrolizumab), and programmed cell death ligand 1 (PD‐L1: monoclonal antibody atezolizumab, durvalumab). The autoimmune adverse events of ICIs are frequent. Dermatologic toxicities are one of the most common immune‐related adverse events (irAEs), occurring in 43%–45% of patients treated with ipilimumab, and approximately 34% of patients treated with nivolumab or pembrolizumab.^1^ Dermatologic toxicities usually occur early in treatment (the first few weeks after the start of treatment), and cases of dermatologic toxicities after the end of treatment have been reported.^2^ The time taken to develop immune‐related cutaneous toxicities has been reported to be shorter for those on combination therapy versus anti‐PD1 monotherapy.^3^

The mechanisms of dermatologic irAEs are not fully understood. However, it is clearly related to T cell activation mediated by inhibiting the PD‐1/PD‐L1 and CTLA‐4 pathway.^4^ ICI‐induced vitiligo may be related to cross‐reactivity against antigens shared by melanoma cells and normal melanocytes.^4^ T‐cell antigens shared between tumor tissue and skin have been identified in patients with NSCLC, and these antigens were able to activate CD4^+^ and CD8^+^ T cells in vitro. In the report by Tanaka *et al*. the serum level of interleukin‐6 increased in nivolumab‐associated psoriasis.^5^ As the PD‐1 blockade augments T‐helper cell 1(Th1)/Th17 signaling pathway it could promote proinflammatory cytokines mediated by Th17 lymphocytes.^6^ Therefore, it is a potential mechanism of ICI‐induced psoriasis.

Most immune‐related cutaneous AEs are mild, and serious cutaneous AEs are rare. However, life‐threatening cases such as drug reaction with eosinophilia and systemic symptoms (DRESS), Stevens‐Johnson syndrome (SJS) and toxic epidermal necrolysis (TEN) have been reported.

Most immune‐related cutaneous AEs respond to treatment, and biologic agents are effective in patients with corticosteroid‐refractory diseases. Increased eosinophils, interleukin‐6 (IL‐6), interleukin‐10 (IL‐10), and immunoglobulin E (IgE) have been reported by Phillips *et al*. to be associated with immune‐related cutaneous adverse events and may be therapeutic targets for immune‐related dermatologic toxicities.^7^

## Clinical manifestation and management of dermatologic toxicities

National Comprehensive Cancer Network (NCCN),^8^ European Society for Medical Oncology (ESMO)^1^ and the Chinese Society of Clinical Oncology (CSCO) have published clinical guidelines on the management of immune‐related adverse events (IrAEs).

Patients need baseline assessment of skin prior to initiating immune checkpoint inhibitors (ICIs). Patients with a history of immune‐related skin disorders, such as bullous pemphigoid, psoriasis, lichenoid reaction, and lupus erythematosus should be assessed by a dermatologist. When a patient has a dermatologic reaction, a detailed history, careful and thorough examination of the skin and mucosa should be taken. Other etiology such as an infection, an adverse effect of another drug and other systemic disease should be excluded when confirming immune‐related dermatologic toxicities.

## Maculopapular rash

Maculopapular rash is one of the most frequent cutaneous irAEs. The severity of maculopapular rash can be classified as three grades according to the Common Terminology Criteria for Adverse Events (CTCAE version 4.02). ESMO guidelines suggest when a rash is diffuse but light and not associated with any additional symptoms, grade 2 would be more appropriate than grade 3. Topical medium‐ to high‐potency corticosteroids, and oral antihistamines are recommended for grade 1 maculopapular rash, and immunotherapy can usually be continued. Systemic corticosteroids (prednisone 0.5–1 mg/kg/day) can be considered for grade 2 maculopapular rash. For grade 2 rashes, ESMO guidelines recommend continuation of ICIs, while the NCCN and CSCO guidelines recommend consideration is given to withholding ICIs. Therefore, for diffuse but mild rashes, the ESMO guidelines are that ICIs should be continued. A dermatologist should be consulted for advice as to whether ICIs should be continued for grade 2 rashes. Patients with grade 3 rashes require discontinuation of ICIs, an urgent dermatology consultation, and treatment with systemic corticosteroids (prednisone 0.5–1 mg/kg/day). Inpatient care can be considered.

It should be noted that maculopapular rash may be an early manifestation of other immune‐related dermatologic toxicities, such as lichenoid reactions, psoriasis, and bullous pemphigoid. Skin biopsies could be considered especially for atypical, severe, and persistent rashes.

## Pruritus

Pruritus is among the most common cutaneous irAEs. A meta‐analysis by Belum *et al*. showed that the incidence of pruritus was 13%–20% in patients treated with nivolumab or pembrolizumab.^9^ Pruritus can occur with a rash or with normal‐appearing skin. The severity of pruritus can be classified as three grades according to CTCAE (version 4.02). Topical medium‐ to high‐potency corticosteroids, oral antihistamines, and topical emollients are recommended for grade 1/2 pruritus, and immunotherapy can usually be continued. Patients with grade 3 pruritus require discontinuation of ICIs, an urgent dermatology consultation, and treatment with systemic corticosteroids (prednisone 0.5–1 mg/kg/day). The addition of γ‐aminobutyric acid agonists (gabapentin or pregabalin) may be helpful. It was reported by Ito *et al*.^10^ that aprepitant (80 mg daily for five days) was effective in the treatment of refractory pruritus caused by nivolumab.

## Lichenoid dermatitis

Lichenoid dermatitis usually occurs after several weeks to months of treatment with ICIs. Clinical manifestation includes typical lichen planus, oral lichen planus, hypertrophic lichen planus, and pruritus. In the study by Schaberg *et al*. histological examination revealed lichenoid interface dermatitis, including a band‐like lymphocytic infiltrate, keratinocyte apoptosis and destruction of the epidermal basal cell layer.^11^ Most patients can be managed by topical glucocorticoids, although some cases need oral corticosteroids, phototherapy, and acitretin.

## Psoriasis

Exacerbation or occurrence of psoriasis is observed in the course of treatment with ICIs.^12^ De novo psoriasis may occur after several months of treatment. Histology showed an intense and confluent epidermal parakeratosis and acanthosis in the report by Sibaud.^4^ Patients can be managed by topical corticosteroids, acitretin, phototherapy, or systemic glucocorticoids. A case of psoriasis induced by pembrolizumab has been previously reported which resolved after treatment with systemic interleukin IL17A blockade (secukinumab).^13^

## Vitiligo

Vitiligo occurs mostly in melanoma patients treated with ICIs. However, patients with other types of cancer have occasionally been reported.^14^ Vitiligo mostly occurs in the first few months after treatment of ICIs. How to manage ICI‐induced vitiligo has not been well established. It was reported by Miyagawa *et al*. that nivolumab‐induced vitiligo was successfully treated with narrowband UVB phototherapy.^15^

## Bullous pemphigoid (BP)

Bullous pemphigoid (BP) has emerged as a rare but serious potential cutaneous AE of checkpoint inhibitor therapy. ICIs can aggravate a pre‐existing BP. BP is more specific to PD‐1/ PD‐L1 pathway inhibitor, although it can also occur during treatment of anti‐CTLA‐4 antibodies.^16^ In the study by Lopez *et al*. BP most frequently occurred within several months after treatment with ICIs. Pruritus and nonspecific rashes presented in the early stages.^17^ Mucosal involvement was rare. Direct immunofluorescence showed linear immunoglobulin G and complement component 3 deposition along basement membrane zone. The treatment approach to checkpoint inhibitor‐induced BP has largely been derived from studies conducted in patients with conventional BP. Development of BP usually requires discontinuation of immunotherapy. ICI‐induced BP are mainly treated with topical or systemic corticosteroids. The treatment of rituximab or omalizumab has been reported in refractory cases.

## Cutaneous capillary endothelial proliferation (CCEP)

Cutaneous capillary endothelial proliferation (CCEP) has been observed in patients treated with camrelizumab. The incidence of CCEP during treatment with camrelizumab monotherapy is reported to be 77.1%. Combination with apatinib or chemotherapy can reduce the incidence of CCEP. CCEP occurs mostly on the skin of face and body surface, and never on the mucosa of respiratory tract and digestive tract. Local treatment to prevent infection is required when there is bleeding. Laser and surgical resection can be considered if necessary. Antibiotics should be given to patients with infection.

## Stevens‐Johnson syndrome (SJS)

Stevens‐Johnson syndrome (SJS) is a rare but serious skin reaction. Clinical manifestations include nonspecific maculopapular rash, blisters, and epidermal necrosis peeling. The oral mucosa is typically involved, and ocular involvement has been reported in patients with SJS. ICIs should be discontinued permanently. An urgent dermatology consultation is required. Intravenous prednisone/methylprednisolone 1 to 2 mg/kg/day, and intravenous immunoglobulin is necessary.

Salati *et al*.^18^ reported a female patient with lung squamous cell carcinoma who developed fever, fatigue, painful oral mucositis, hemorrhagic crusts on lips, and maculopapular rashes involving lower limbs, palms and soles after treatment of nivolumab. Systemic corticosteroid treatment with methylprednisolone 1 mg/kg and nutritional support were initiated and her symptoms gradually improved.

## Toxic epidermal necrolysis (TEN)

Toxic epidermal necrolysis (TEN) is a life‐threatening skin disorder characterized by a blistering and peeling of the skin. Mucous membranes are usually involved. The involved skin is ≥30% of the total body surface area. ICIs therapy should be immediately discontinued. Patients require hospitalization. Fluid and electrolyte management as well as intensive care are necessary. High dose corticosteroids (methylprednisolone 1–2 mg/kg daily) and intravenous immunoglobulin should be administered. In severe or steroid‐unresponsive cases, the addition of other agents such as infliximab, mycophenolate mofetil, or cyclosporin may be considered.

Vivar *et al*.^19^ reported that a female patient with melanoma developed TEN during treatment with nivolumab. Nivolumab was withheld. A biopsy demonstrated mild interface dermatitis with rare necrotic keratinocytes. Immunohistochemical analysis demonstrated CD8+ cells aggregated at the dermal‐epidermal junction and epidermal exocytosis of CD8+ cells. PD‐L1 expression was markedly increased in the epidermis. Prednisone 1 mg/kg, infliximab, intravenous immunoglobulin and broad‐spectrum antibiotics were administered. However, the patient eventually died of septic shock and multisystem organ failure.

## Drug reaction with eosinophilia and systemic symptoms (DRESS)

DRESS is a drug‐induced hypersensitivity reaction associated with multiorgan involvement. It is characterized by fever, rash, eosinophilia, and the liver is frequently involved. ICIs should be discontinued if DRESS occurs. Treatment with high dose systemic corticosteroids and supportive care should be given. It is a rare but potentially fatal adverse event.

Mirza *et al*.^20^ reported that a male melanoma patient presented with fever, headache, and rash over his face, back and chest after receiving treatment with ipilimumab and nivolumab. A complete blood count showed eosinophil count of 1.41 × 109/L. The patient was clinically diagnosed with DRESS and treated with intravenous methylprednisolone 100 mg daily. The rash, eosinophilia and transaminitis improved.

## Sweet syndrome

Sweet syndrome, also called acute febrile neutrophilic dermatosis, was first described by Robert Douglas Sweet in 1964. It is characterized by abrupt onset of painful erythematous plaques or nodules, and predominantly neutrophilic dermal infiltrate without leukocytoclastic vasculitis. It has a dramatic response to systemic corticosteroids.

Adler *et al*.^21^ reported a male melanoma patient who presented with multiple tender, purpuric plaques on his left hand, generalized malaise and fever after receiving four doses of ipilimumab. A complete blood count showed leukocytosis. Skin biopsy revealed an intense neutrophilic dermal infiltrate without leukocytoclastic vasculitis. Discontinuation of ipilimumab and treatment of oral prednisolone and topical corticosteroid resulted in complete resolution.

## Immune checkpoint inhibitor rechallenge

For maculopapular rash or pruritus, consider resuming ICIs after symptoms have resolved to ≤ grade 1. ICIs should be permanently discontinued for severe life‐threatening dermatologic toxicities such as SJS, TEN, and DRESS.

## Dermatologic toxicities and clinical efficacy of ICIs

Vitiligo seems to be associated with better clinical outcomes in melanoma patients who received anti‐PD‐1 monoclonal antibody.^22^ It has been reported that the repigmentation of vitiligo could be a marker of disease progression of melanoma.^23^, ^24^ In a prospective cohort study undertaken by Berner *et al*. 73 patients with advanced NSCLC who received nivolumab or pembrolizumab were recruited and of these, 25 patients (34.2%) developed immune‐related dermatologic toxicities. The incidence of immune‐related dermatologic toxicities in patients with complete/partial response was higher than that in patients with stable/progressive disease (68.2% vs. 19.6%).^25^ In the study by Feng *et al*. patients with primary hepatic carcinoma with CCEP tended to have a higher response rate during camrelizumab monotherapy.^26^

In conclusion, although the incidence of immune‐related adverse skin reactions is high, most are mild and self‐limiting. Only a few severe or life‐threatening AEs have been previously reported. A dermatology consultation is needed to diagnose and manage adverse events. Patient education, early identification and appropriate treatment are crucial in the management of immune‐related dermatologic toxicities.

## Disclosure

The authors have no potential conflicts of interest to disclose.
